# Stress, well-being, and optimism in Portuguese youth: how do gender and age shape mental health and eating quality

**DOI:** 10.3389/fpsyg.2026.1593283

**Published:** 2026-03-13

**Authors:** Adelinda Araújo Candeias, Adriana Simões Félix, Edgar Galindo

**Affiliations:** 1 Department of Medical Sciences and Health, School of Health and Human Development, Comprehensive Health Research Center, University of Evora, Évora, Portugal; 2 Comprehensive Health Research Center, University of Evora, Évora, Portugal

**Keywords:** developmental differences, eating quality, gender differences, mental health, optimism, stress, well-being

## Abstract

Stress has been consistently linked to changes in eating behaviors and psychological well-being among adolescents and young adults, yet evidence remains fragmented regarding how gender, developmental stage, and psychological resources jointly shape these outcomes. Grounded in stress–coping models, self-regulation theories, and a life-span developmental perspective, this study examines age- and gender-related differences in stress, well-being, optimism, and eating quality within a post-lockdown context. A cross-sectional study was conducted with 951 Portuguese adolescents and young adults aged 15–26 years. Data were collected across three post-lockdown periods (2020–2022) and aggregated after confirming negligible time-related effects (*η*^2^ ≤ 0.010). Validated self-report measures assessed perceived stress, well-being, optimism, and eating quality. Main and interaction effects of gender and developmental stage were examined using ANOVA and MANOVA. Significant main effects of gender and age were found for stress, well-being, and optimism. Female participants reported higher stress and lower well-being and optimism than males, with small to moderate effect sizes (*η*^2^ ≈ 0.01–0.04). Young adults displayed lower stress and higher well-being and optimism than adolescents (*η*^2^ ≈ 0.02–0.05). Eating quality showed no significant gender differences and limited age variation. Interaction effects were modest. Higher optimism was consistently linked to more favorable well-being and eating profiles. Findings support an integrative model in which stress-related outcomes are shaped by gender, developmental stage, and psychological resources. Interventions should strengthen optimism, self-regulation, and stress management using developmentally tailored approaches.

## 1 Introduction

Adolescence and early adulthood represent sensitive periods in which stress is consistently linked to changes in eating behaviors and psychological well-being; nevertheless, evidence remains fragmented regarding how gender, developmental stage, and psychological resources jointly influence these outcomes. In particular, understanding whether stress-related eating patterns reflect universal mechanisms or gendered pathways remains critical, given that emotional regulation capacities, coping strategies, and self-related resources continue to consolidate across these transitions. Moreover, the post-lockdown period has heightened concerns around youth mental health and lifestyle behaviors, reinforcing the need for context-sensitive evidence that integrates developmental and psychosocial perspectives.

Gender has consistently emerged as a relevant moderator of the stress–eating relationship. Early research indicated that women under stress were more likely than men to increase consumption of high-calorie and high-fat foods (Grunberg and Straub, 1992). More recent studies show that young women often report higher stress levels than their male counterparts, potentially due to differences in coping styles, emotional processing, and social expectations (Shamsuddin et al., 2013). This gendered vulnerability in emotional responding has also been observed in Portuguese youth, with evidence suggesting differentiated emotional profiles and stress-related adjustment patterns across gender (Candeias, 2025). In academic contexts, females tend to experience greater stress in response to academic challenges, while males and females differ significantly in how stress translates into eating behaviors (Herndon and Moore III, 2002; Mohamed et al., 2020).

These differences are frequently interpreted through gendered stress–coping pathways. Women are more likely to internalize stress, manifesting anxiety or depressive symptoms, whereas men tend to externalize stress through irritability or aggression (Buecker et al., 2023; Roslan et al., 2017). Such patterns may help explain why women are more prone to emotional or restrictive eating behaviors, while men may exhibit overeating or place less emphasis on dietary health (Bogg and Roberts, 2004; Carver and Scheier, 2014). Evidence from non-Western contexts reinforces this differentiation; for instance, a recent study conducted in Pakistan showed that males predominantly coped with academic stress through overeating, whereas females displayed more heterogeneous responses, ranging from overeating to reduced intake (Shah et al., 2024). Collectively, these findings indicate that gender is not merely a demographic variable, but a theoretically meaningful dimension shaping stress appraisal, coping, and eating behaviors.

### 1.1 Developmental transitions: adolescence to young adulthood

Age and developmental stage constitute another critical dimension in understanding stress-related eating behaviors and well-being. Adolescents and young adults face qualitatively different stressors that may differentially shape psychological adjustment and health behaviors. Adolescents (approximately 15–18 years) are primarily confronted with academic performance and peer-related pressures, whereas young adults (19–25 years) increasingly face challenges associated with autonomy, career development, financial responsibility, and intimate relationships. Research suggests that younger adolescents may display greater resilience, while stress levels tend to increase during the transition to young adulthood as responsibilities accumulate (Buecker et al., 2023; Mascherini et al., 2021).

These developmental differences are particularly relevant given the long-term health implications of stress-related eating. Stress-induced eating behaviors have been associated with increased risk of overweight and obesity in adulthood (Berridge et al., 2010), and similar psychophysiological mechanisms appear to contribute to the rising prevalence of obesity in childhood and adolescence (Pervanidou and Chrousos, 2016). Because eating habits established during adolescence often persist into adulthood (Alberga et al., 2012), this developmental period represents a critical window for long-term physical and mental health outcomes (Ramón-Arbués et al., 2019). Nevertheless, evidence in younger populations remains less conclusive than in adults. A meta-analysis by Hill et al. (2018) showed that stress is associated with unhealthy eating behaviors across childhood and adolescence, while its association with healthy eating emerges primarily in older age groups, underscoring the need to further examine developmental moderators.

### 1.2 Psychological resources: optimism and well-being

Beyond demographic and developmental factors, psychological resources play a central role in shaping responses to stress. Personality traits, emotional states, and dispositional optimism have all been linked to stress-related eating behaviors. Individuals high in neuroticism are more prone to emotional or comfort eating under stress (Macht, 2008), whereas extraverted individuals tend to make healthier food choices, possibly due to greater social engagement around meals (Keller and Siegrist, 2015).

Optimism has emerged as a particularly relevant protective factor. Optimistic individuals are more likely to engage in health-promoting behaviors and to buffer the impact of stress on eating quality and psychological well-being (Bouchard et al., 2018; Shaheed et al., 2022). At the same time, overly optimistic perceptions may lead to underestimation of health risks, highlighting the importance of examining optimism in conjunction with stress and well-being rather than in isolation (Carver and Scheier, 2014). Recent nutritional and public health studies further support the inclusion of psychological resources in models of eating behavior, emphasizing that stress-related dietary patterns are embedded in broader self-regulatory and psychosocial processes (Yılmaz et al., 2020; Yılmaz et al., 2024).

### 1.3 Integrative theoretical framework

The empirical evidence reviewed above can be coherently interpreted through an integrative theoretical framework combining stress–coping models, self-regulation theories, and a life-span developmental perspective. From a stress–coping viewpoint, stress is conceptualized as a transactional process arising from the interaction between environmental demands and individual appraisal and coping resources. Within this framework, eating behaviors can be understood as coping strategies aimed at regulating emotional states under stress.

Self-regulation theories further emphasize that stress taxes executive functioning and emotional regulation, potentially undermining individuals’ capacity to maintain adaptive behaviors such as healthy eating and psychological balance. Psychological resources such as optimism may buffer these effects by shaping stress appraisals, sustaining goal-directed behavior, and supporting emotional regulation. A life-span developmental perspective suggests that these processes are not static, but vary across adolescence and young adulthood due to ongoing maturation of self-regulatory systems and changing psychosocial demands. Gendered socialization and coping patterns further modulate these dynamics, influencing how stress is experienced and managed across developmental stages.

### 1.4 Pandemic context and the Portuguese setting

The coronavirus disease 2019 (COVID-19) pandemic constituted an unprecedented stressor that amplified existing vulnerabilities in young populations. International studies consistently report that pandemic-related stress and social restrictions were associated with increased food intake, greater reliance on processed and comfort foods, and disrupted eating routines (Renzo et al., 2020; Marty et al., 2021; Flaudias et al., 2020). A large cross-national study identified distinct behavioral profiles, including a less healthy cluster characterized by affect-driven eating and a healthier cluster more prevalent among highly educated individuals, highlighting the role of psychosocial resources and social inequalities (Lamy et al., 2022).

Portugal represents a particularly informative context in which to examine these dynamics. Recent data indicate high levels of stress, anxiety, and depressive symptoms among Portuguese adolescents and young adults, placing Portugal among the most affected countries in the European Union (OECD, 2022; Carvalho et al., 2024). During the pandemic, Portuguese young adults reported lower quality of life, optimism, and well-being compared to older age groups (Candeias et al., 2021), while adolescents also experienced marked declines in mental health indicators (Guedes et al., 2023). In this sense, Portugal offers a relevant post-pandemic context to examine how stress, psychological resources, and eating behaviors intersect during critical developmental stages.

### 1.5 The present study: research questions and hypotheses

Taken together, existing research highlights the importance of stress in shaping eating behaviors and well-being, while identifying gender, developmental stage, and psychological resources as key moderators. However, these dimensions are often examined in isolation. There remains a need for integrative studies that simultaneously consider stress, well-being, optimism, and eating quality within a unified theoretical framework that explicitly incorporates both gender and developmental differences, particularly in contexts marked by heightened psychosocial vulnerability.

Accordingly, the present study addresses the following research questions (RQ):

*RQ1*: How does gender influence stress levels, well-being, optimism, and eating quality among Portuguese youth?

*RQ2*: What differences exist across developmental stages (middle adolescence, late adolescence, and young adulthood) in stress, well-being, optimism, and eating quality?

*RQ3*: Does the interaction between gender and age explain additional variance in stress, well-being, optimism, and eating quality beyond their independent effects?

Guided by the integrative framework described above, these questions are further articulated through the following theory-driven hypotheses (H1 to H4):

*H1* (Gender differences): Female participants will report higher perceived stress and lower eating quality than male participants, alongside differences in well-being and optimism consistent with gendered stress–coping patterns.

*H2* (Developmental differences): Differences will emerge across developmental stages, with young adults reporting higher stress levels but also higher optimism and well-being compared to middle and late adolescents, reflecting age-related changes in self-regulatory and coping resources.

*H3* (Psychological resources): Higher levels of optimism will be positively associated with greater well-being and better eating quality, particularly under conditions of elevated stress.

*H4* (Interaction effects): Gender and age will interact in explaining variations in stress, well-being, optimism, and eating quality, such that developmental differences manifest differently for male and female participants.

## 2 Materials and methods

### 2.1 Sample

This study employs a cross-sectional design with data collected at three distinct time points, aggregated into a single sample. This approach was chosen to maximize sample size and ensure a more robust representation of the population during the challenging post-lockdown periods (Jones and Johnston, 2017). The recruitment strategy remained consistent across all phases, utilizing institutional mailing lists and social media outreach to target Portuguese youth full-time, non-employed aged 15–26 years (*N* = 830,317; FFMS, 2023). A convenience, non-probability sampling approach was employed, utilizing online dissemination and an electronic platform for data collection due to their accessibility. The sample comprised 951 participants (1.15% of the total population), including 364 (38.28%) from secondary schools and 587 (61.72%) from higher education institutions, representing all regions of the country. Regarding gender distribution, 698 (73.40%) participants were female. While this imbalance reflects a common trend in online health surveys, it is acknowledged as a potential participation bias, and results should be interpreted with caution regarding their generalizability to male youth. Data collection occurred in three phases: (1) 17 June to 2 August 2020; (2) 5 March to 7 April 2021; and (3) 15 March to 15 April 2022.

To assess the feasibility of aggregating data from the three collection waves (2020–2022), a series of one-way analyses of variance (ANOVAs) was conducted. Although statistically significant differences across time points were observed for well-being [*F* (2, 948) = 4.74, *p* = 0.009], optimism [*F* (2, 948) = 3.72, *p* = 0.024], and chronic stress [*F* (2, 948) = 3.07, *p* = 0.047], the corresponding effect sizes were negligible (all *η*^2^ ≤ 0.010). According to Cohen (1992) guidelines, these values indicate that less than 1% of the variance in these psychological constructs is attributable to the time of data collection. Consequently, the observed differences were considered to have limited practical significance, supporting the decision to aggregate the data and treat the sample as a single cross-sectional dataset.

Importantly, data collection occurred during successive post-lockdown periods, which together represent a unique and historically bounded context of heightened psychosocial stress, social restriction, and academic disruption. Rather than reflecting stable temporal trends, the three waves capture variations within an exceptional situational context that is unlikely to be replicated under normative conditions. From this perspective, aggregating the data allows the analysis of a broader and more robust snapshot of stress-related psychological and behavioral responses under constrained circumstances. Accordingly, the findings should be interpreted as characterizing patterns of vulnerability and resilience during an extraordinary period of social disruption, rather than as indicators of long-term developmental or temporal change.

The study adopted a cross-sectional design with comparisons by gender and developmental stage (middle adolescence, late adolescence, and young adulthood), allowing the examination of main and interaction effects in line with the proposed research questions.

Based on these statistical results, the Portuguese organization of formal education for youth (Compulsory education until 18 years old, Education System Basic Law, 46 from 1986), and in the pediatrics classification from the American Association of Pediatrics of this period of age (Allen & Waterman, 2024) we organize the sample in three age groups:

Group 1 – Middle Adolescents (G1): 15 to 18 years (*N* = 387; *M* = 16.930; SD = 0.883);
Group 2 – Late Adolescents (G2): 19 to 22 years (*N* = 398; *M* = 20.650; SD = 1.086);
Group 3 – Young Adults (G3): 23 to 26 years (*N* = 166; *M* = 23.730; SD = 0.986).

Table 1 details the demographic distribution. The youngest cohort (G1) primarily consists of secondary students living with parents; G2 is largely composed of undergraduates in independent or shared housing; and G3 represents those in postgraduate studies or transitioning to independent households. Household was characterized in terms of household size, operationalized as the number of persons living in the household and grouped into five categories (1, 2, 3, 4, and 5 or more persons), as reported in Table 1. Participants were geographically distributed across mainland Portugal and the islands (North: 5.0%; Center: 57.7%; South: 29.5%; Islands: 5.3%).

**Table 1:** Participants distribution by household size (number of persons), age group, and gender.

Number of people in the household	Age group	Sex		*N*	Mean age (SD)
		Male	Female		
1	1 (15 to 18 years)	4	7	11	17.00 (0.82)
	2 (19 to 22 years)	6	13	19	20.83 (1.17)
	3 (23 to 26 years)	5	6	11	24.40 (1.52)
Subtotal	–	15 (5.92%)	26 (3.7%)	41 (4.31%)	–
2	1 (15 to 18 years)	5	28	33	17.20 (0.84)
	2 (19 to 22 years)	11	43	54	20.91 (0.94)
	3 (23 to 26 years)	11	21	32	23.90 (1.12)
Subtotal	–	27 (10.67%)	92 (13.18%)	119 (12.51%)	–
3	1 (15 to 18 years)	28	94	122	16.96 (0.85)
	2 (19 to 22 years)	30	115	145	20.80 (1.05)
	3 (23 to 26 years)	19	35	54	23.68 (1.21)
Subtotal	–	77 (30.43%)	244 (34.96%)	321 (33.75%)	–
4	1 (15 to 18 years)	46	120	166	16.93 (0.85)
	2 (19 to 22 years)	30	95	125	20.71 (1.06)
	3 (23 to 26 years)	19	34	53	23.50 (1.21)
Subtotal	-	95 (37.55%)	249 (35.67%)	344 (36.17%)	–
5 or more	1 (15 to 18 years)	22	33	55	16.68 (0.84)
	2 (19 to 22 years)	12	43	55	20.75 (1.06)
	3 (23 to 26 years)	5	11	16	24.00 (1.12)
Subtotal	–	39 (15.42%)	87 (12.46%)	126 (13.25%)	–
Total	–	253 (100%)	698 (100%)	951 (100%)	–

Regarding lifestyle variables, more than half of the participants (53.5%) reported regular sports engagement, with the most frequent commitments being 3 h (10.6%), 2 h (9.1%), or 1 h (7.8%) per week. Internet usage was high among the respondents; notably, a significant portion of the sample spent between 5 and 8 h online daily (13.2% spent 5 h, 12.5% spent 6 h, and 12.2% spent 8 h). Concerning dietary habits, 43.6% of the participants maintained a regular frequency of four meals per day.

### 2.2 Instruments

The research utilized a multidimensional protocol combining psychosocial scales and a socio-demographic survey.

#### 2.2.1 Psychosocial scales

1. Chronic Stress Test: A 7-item scale (Reschke, 2011) evaluating chronic stress on a 4-point Likert scale (7 to 28 points). Scores under 17 suggest elevated levels of chronic stress. The original version (Reschke and Schröder, 2010) demonstrated strong psychometric properties, with a test–retest reliability of 0.812 and a Cronbach’s alpha of 0.743. Adaptation studies for the Portuguese population, confirmed its reliability and factorial structure.
2. General Index of Well-being: A 25 items instrument (Massé et al., 1998) assessing six dimensions: self-esteem, balance, social involvement, sociability, self-control, and happiness. Responses are rated on a 5-point Likert scale, higher scores reflect greater perceived psychological well-being. Portuguese versions showed strong internal consistency (alpha between 0.67 and 0.89).
3. Optimism Scale: A 4-item unidimensional scale (Barros, 1998) measuring overall optimism on a 5-point Likert scale (maximum possible score of 20). The Portuguese version demonstrated adequate reliability (alpha = 0.76) (Barros, 1998).

#### 2.2.2 Measurement of eating quality

The “Eating Quality” variable was derived from the Lifestyle Checklist (Guerra, 2004), specifically focusing on self-reported dietary changes compared to the pre-pandemic period.

Development of the Index: Given the open-ended nature of the qualitative data (e.g., “Has your diet changed? Justify”), an Eating Quality Index was developed using a secondary analysis approach. To translate these qualitative justifications into a 3-level ordinal scale (0: Lower Quality; 1: Same Quality; 2: Higher Quality), we employed the following rigorous procedure:

1 Coding Criteria: A panel of three experts (Nutrition, Health Psychology, and Education) established coding categories based on the Portuguese Food Wheel Guide, the official national food-based dietary guideline from Portuguese Directorate-General of Health (DGS Direção-Geral da Saúde, 2016).

- *Lower Quality (0):* Reports of increased intake of ultra-processed foods, skipping main meals, or reduction in fruit/vegetable consumption.
- *Same Quality (1):* Reports of maintaining previous habits or changes with no nutritional impact.
- *Higher Quality (2):* Reports of increased water intake, regular meal schedules, or higher consumption of fresh produce.

2 Expert Consensus: The panel performed an initial individual classification of a subset of responses, followed by a group discussion to resolve discrepancies. The final inter-rater agreement reached 85% (Cohen’s Kappa ≈ 0.79), ensuring high reliability in the translation of qualitative perceptions into quantitative data.
3 Validity Argument: Although this index is based on self-perception, it serves as a robust screening measure of perceived behavioral shifts in lifestyle, which is highly relevant for large-scale psychosocial studies.

#### 2.2.3 Socio-demographic questionnaire

A Sociodemographic Questionnaire, designed specifically for this study, aimed to gather essential information to characterize the sample, including details such as age, gender, family composition, place of residence, current course of study, and academic year.

### 2.3 Procedure/ethics approval

This research is a part of the broader project titled “Well-being and Quality of Life,” approved by the Ethical Committees from University of Évora number 24097. The research adhered to the Declaration of Helsinki and the ethical framework for web-based research (Franzke et al., 2019).

To ensure methodological rigor and transparency in online data collection, the study protocol adhered to the Checklist for Reporting Results of Internet E-Surveys CHERRIES, Eysenbach (2012) guidelines. In line with CHERRIES recommendations, the protocol ensured transparent reporting of recruitment channels, standardized informed consent procedures, and procedures to reduce the likelihood of duplicate entries. The electronic survey was disseminated through institutional mailing lists and social media platforms (Facebook, Twitter/X, WhatsApp, and Instagram) across the three data collection phases described above.

Upon accessing the survey, participants were presented with an information sheet and an informed consent form on the initial page; participation was entirely voluntary, anonymous, and non-remunerated. All respondents maintained the autonomy to withdraw from the study at any point without the need for justification.

To guarantee the integrity of the database and prevent multiple submissions, a technical control measure was implemented requiring a Google sign-in to access the form. While it is acknowledged that this requirement may introduce a minor participation bias by excluding individuals without an active Google account, this methodological choice was prioritized to mitigate the risk of duplicate responses and reinforce the overall reliability of the findings. To maintain participant confidentiality, no personal identification data were requested or recorded. The collected responses were exported from the Google Forms platform into Excel and subsequently converted into IBM SPSS Statistics (version 28) for processing. All data were stored in a secure, protected database with access restricted exclusively to the research team.

### 2.4 Data analysis

The study adopted a cross-sectional design with comparisons by gender and developmental stage (middle adolescence, late adolescence, and young adulthood), allowing the examination of main and interaction effects in line with the research questions and hypotheses.

Descriptive statistics were computed for all variables. Gender differences (RQ1/H1) and age-group differences (RQ2/H2) were examined using group comparisons, and interaction effects between gender and age (RQ3/H4) were tested. Associations between optimism, well-being, stress, and eating quality (H3) were explored using correlational analyses.

Inferential analyses were conducted using ANOVA and multivariate analysis of variance (MANOVA). Univariate normality was assessed using the Kolmogorov–Smirnov and Shapiro–Wilk tests, revealing some deviations from normality for optimism, stress, and eating quality; given the large sample size (N = 951), these deviations were considered acceptable (Marôco, 2014; Tabachnick & Fidell, 2019). Homogeneity of variances was examined with Levene’s test, and equality of covariance matrices with Box’s M test. As Box’s M was significant, Pillai’s Trace was used as the primary multivariate statistic due to its robustness to assumption violations.

Factorial ANOVA was applied to test main and interaction effects, and MANOVA examined the combined effects of gender and age on eating quality, well-being, optimism, and stress. Effect sizes were calculated and interpreted following established guidelines (Cohen, 1988; Espirito Santo and Daniel, 2017), and post-hoc comparisons were performed using Tukey’s test when appropriate (Keselman and Rogan, 1977). Statistical significance was set at *α* = 0.05 for all analyses (Schlarb et al., 2017).

## 3 Results

Descriptive statistics and intercorrelations are presented in Table 2. The analysis aimed to identify overall association patterns among psychosocial and lifestyle variables.

**Table 2:** Descriptive statistics and intercorrelation matrix among study variables.

Variables	Mean (SD)	1	2	3	4	5	6	7	8	9
1. Age Group	1.77 (0.73)	1								
2. Household	3.42 (1.01)	−0.141^**^	1							
3. Eating Quality	1.09 (0.38)	−0.081^*^	0.018	1						
4. Well-being	83.90 (17.68)	0.017	0.039	0.089^**^	1					
5. Chronic Stress	1.41 (0.49)	−0.145^**^	0.046	0.048	−0.422^**^	1				
6. Optimism	14.04 (3.66)	0.018	0.008	0.060	0.754^**^	−0.333^**^	1			
7. Daily meals	3.92 (0.85)	0.009	0.036	0.035	0.136^**^	−0.029	0.102^**^	1		
8. Weekly sports hours	3.64 (5.50)	−0.068	−0.025	0.064	0.189^**^	−0.031	0.171^**^	0.096^**^	1	
9. Daily internet hours	8.27 (27.85)	0.011	−0.022	−0.012	−0.036	0.006	−0.025	−0.009	−0.038	1

*The correlation is significant at the 0.05 level (2 ends). **The correlation is significant at the 0.01 level (2 ends).

A clear psychosocial core emerged. Well-being showed a strong positive association with optimism (*r* = 0.754, *p* < 0.01) and a moderate negative association with chronic stress (*r* = −0.422, *p* < 0.01). Optimism was also moderately and negatively related to stress (*r* = −0.333, *p* < 0.01), indicating that higher well-being and optimism tend to co-occur with lower stress. Associations involving eating quality were statistically significant but small, including a weak positive correlation with well-being (*r* = 0.089, *p* < 0.01) and a slight negative association with age group (*r* = −0.081, *p* < 0.05). Lifestyle behaviors showed small yet consistent effects: daily meals and weekly sports hours were weakly associated with higher well-being and optimism (all rs between 0.10 and 0.19, *p* < 0.01). Daily internet use showed no meaningful associations.

Age group was weakly and negatively related to household size (*r* = −0.141, *p* < 0.01), with younger participants more likely to live in larger households. Overall, correlations indicate strong links among psychological variables and modest associations with lifestyle factors, providing contextual support for subsequent group analyses.

### 3.1 Differences between groups

Group differences were examined using ANOVAs, and MANOVA was conducted to characterize the overall multivariate pattern across well-being, optimism, stress, and eating quality (Tables 3, 4). Descriptive results are presented as means (M) and standard deviations (SD). For each ANOVA, we report the F statistic, effect sizes (*η*^2^), and Tukey’s significant difference *post hoc* comparisons when applicable.

**Table 3:** Group differences by gender.

Measure	Male		Female		*F*	η^2^
	*M*	SD	*M*	SD		
Eating quality	1.09	0.32	1.09	0.40	0.014	0.000
Well-being	90.14	18.29	81.64	16.90	44.950	0.045
Optimism	14.85	3.68	13.75	3.61	17.064	0.018
Stress	14.68	4.61	17.01	4.84	44.086	0.044

**Table 4:** Differences by age group.

Measure	Middle adolescents		Late adolescents		Young adults		*F*	η^2^	Tukey
	*M*	SD	*M*	SD	*M*	SD			
Eating quality	1.13	0.47	1.06	0.33	1.06	0.24	4.082*	0.009	G2 < G1
Well-being	84.76	18.54	81.71	16.80	87.16	17.08	6.409**	0.013	G1 > G2 < G3
Optimism	14.21	3.94	13.58	3.43	14.73	3.40	6.622**	0.014	G1 > G2 < G3
Stress	16.97	5.03	16.41	4.83	15.01	4.44	9.533***	0.020	G1 > G3 < G2

As shown in Table 3, gender differences were significant for well-being, optimism, and stress. Males reported higher well-being, *F* (1, 949) = 44.95, *p* < 0.001, *η*^2^ = 0.045, and optimism, *F* (1, 949) = 17.06, *p* < 0.001, *η*^2^ = 0.018, while females reported higher stress, *F* (1, 949) = 44.09, *p* < 0.001, *η*^2^ = 0.044. These effects ranged from small to moderate, with the largest effects observed for well-being and stress. No gender differences were found for eating quality, *F* (1, 949) = 0.01, *p* = 0.906.

Age-group effects (Table 4) were statistically significant but small in magnitude. Well-being, *F* (2, 948) = 6.41, *p* < 0.01, *η*^2^ = 0.013, and optimism, *F* (2, 948) = 6.62, *p* < 0.01, *η*^2^ = 0.014, tended to increase with age, while stress decreased, *F* (2, 948) = 9.53, *p* < 0.001, *η*^2^ = 0.020. Differences in eating quality were minimal, *F* (2, 948) = 4.08, *p* < 0.05, *η*^2^ = 0.009.

The MANOVA showed significant multivariate effects for gender, Pillai’s Trace = 0.048, *F* (4, 942) = 11.97, *p* < 0.001, and age group, Pillai’s Trace = 0.031, *F* (8, 1886) = 3.72, *p* < 0.001, with small effect sizes. No significant gender × age interactions were observed, *p* = 0.160.

Taken together, the results describe a coherent and interpretable pattern. Gender emerges as a meaningful factor for psychosocial well-being, optimism, and stress, with females reporting higher stress and lower well-being, while eating quality remains largely stable across genders. Age-related differences are present but modest, reflecting gradual developmental changes rather than marked group contrasts. Across analyses, eating quality appears less sensitive to demographic variation than psychosocial indicators.

## 4 Discussion

The present findings are interpreted within an integrative framework combining stress–coping models, self-regulation processes, and a life-span developmental perspective. This multidimensional approach provides a coherent explanation for the observed gender- and age-related variations in stress, well-being, optimism, and eating quality among Portuguese adolescents and young adults, while remaining consistent with the theoretical assumptions outlined in the Introduction.

Consistent with H1, significant gender differences emerged across most psychosocial indicators. Female participants reported higher levels of perceived stress and lower levels of well-being and optimism compared to males. These findings align with previous research suggesting gender-differentiated stress–coping pathways, whereby females are more likely to internalize stress, manifesting greater emotional burden and reduced subjective well-being, while males may rely on externalizing or defensive coping strategies that preserve higher levels of optimism (Shamsuddin et al., 2013). Importantly, although these differences were statistically significant, effect sizes were small to moderate, indicating substantial overlap in the post-pandemic experiences of young men and women. This suggests that gender acts as a meaningful but non-deterministic factor in shaping stress-related outcomes.

Notably, no gender differences were observed in eating quality. This finding suggests that changes in dietary habits during the post-lockdown period affected Portuguese youth transversally, regardless of gender. Such patterns are likely attributable to shared contextual influences, including disruptions in daily routines, altered food availability, and increased reliance on family-based eating environments during lockdown, rather than to gender-specific sociocultural pressures.

Developmental differences across age groups provide further support for H2 and the life-span developmental framework. Young adults (23–26 years) demonstrated better psychological adjustment than middle and late adolescents, reporting higher levels of well-being and optimism alongside lower stress. From a developmental perspective, this pattern may reflect the consolidation of more effective self-regulation strategies and coping resources during the transition into adulthood, which enhance resilience in the face of environmental stressors (Buecker et al., 2023).

In contrast, middle adolescents (15–18 years) reported the highest stress levels. This finding is consistent with the literature highlighting adolescence as a period of heightened vulnerability, marked by academic transitions, identity formation, and strong dependence on peer interactions—all of which were significantly disrupted during the pandemic. Interestingly, this younger group also reported slightly higher eating quality, which may be explained by the continued influence of the family environment and parental supervision over food choices, an influence that tends to diminish as autonomy increases in young adulthood.

In line with H3, the strong positive association between well-being and optimism underscores the role of optimism as a central psychological resource. Rather than functioning solely as an outcome of well-being, optimism appears to buffer the negative effects of chronic stress by shaping stress appraisal and supporting emotional regulation. This finding reinforces the theoretical positioning of optimism within stress–coping and self-regulation frameworks and supports the relevance of interventions aimed at fostering positive future-oriented expectations.

Regarding H4, the observed patterns suggest that age and gender jointly shape vulnerability and resilience processes, even when interaction effects are modest. This reinforces the notion that stress-related outcomes during adolescence and young adulthood cannot be fully understood without considering the intersection of developmental stage and gendered coping pathways.

Importantly, the stability of findings across the three collection waves (2020–2022), combined with negligible effect sizes for time-related differences, suggests that Portuguese youth were embedded in a context of sustained psychosocial stress during the post-lockdown period. Rather than reflecting transient fluctuations, the data capture adaptation processes within an exceptional and historically bounded context in which pandemic-related stress became a relatively stable environmental condition. This interpretation is consistent with recent evidence documenting prolonged mental health vulnerability among young populations in similar post-pandemic contexts (Yılmaz et al., 2020; Yılmaz et al., 2024).

### 4.1 Limitations

Several limitations should be acknowledged. A key strength of this study lies in the use of data collected across successive post-lockdown periods, capturing a unique context of heightened psychosocial stress. Although small statistical differences across collection waves were observed, their negligible effect sizes supported the aggregation of the data into a single cross-sectional sample, enabling a robust analysis of gender and developmental differences. Nevertheless, the findings should be interpreted as reflecting responses to a specific contextual moment rather than stable developmental or temporal trends, limiting generalizability beyond post-pandemic conditions.

The sample also presented a notable gender imbalance, with a predominance of female participants (73.4%). While this pattern is common in online health-related surveys, it may constrain the generalization of findings to male populations, particularly given well-documented gender differences in stress and coping pathways.

Additionally, the exclusive reliance on self-report measures may introduce social desirability and recall biases. Although such measures are appropriate for assessing subjective constructs such as optimism and well-being, future research would benefit from incorporating objective indicators or multi-informant approaches, particularly for eating quality.

Finally, although data were collected across three waves, the cross-sectional design precludes causal inferences and limits the ability to examine individual trajectories of change. Longitudinal studies are needed to better understand how stress, optimism, well-being, and eating behaviors evolve across developmental stages. Moreover, as the sample consisted exclusively of students, caution is warranted when generalizing findings to other youth populations, such as those not in education, employment, or training (NEETs).

## 5 Conclusion

The present study highlights meaningful developmental differences in stress, well-being, optimism, and eating behaviors among Portuguese adolescents and young adults, supporting life-span developmental models that conceptualize adolescence and young adulthood as distinct stages characterized by different psychological, social, and behavioral challenges (Buecker et al., 2023; Mascherini et al., 2021; Shaheed et al., 2022; FFMS, 2023).

From an applied perspective, the findings underscore the need for developmentally sensitive interventions. For adolescents, strategies should prioritize emotional regulation, stress management, and resilience-building, with strong involvement of families to reinforce supportive environments and promote healthy eating behaviors. Addressing academic and social pressures may be particularly important for reducing anxiety and depressive symptoms, which appear more pronounced among female adolescents (Shamsuddin et al., 2013; Zaheer & Khan, 2022).

For young adults, interventions should focus on fostering autonomy, self-efficacy, and adaptive coping with the challenges of early adulthood, including career development and relational stressors. Programs aimed at strengthening optimism may be particularly beneficial, given its strong association with well-being and its protective role against stress-related psychological difficulties (Shaheed et al., 2022). Supporting sustainable and independent healthy eating practices also remains a key priority as young adults transition away from family-regulated environments.

Despite growing evidence, inconsistencies across studies indicate that important questions remain open. Future research should further explore contextual and socio-cultural factors shaping these developmental outcomes, using longitudinal and mixed-methods approaches to capture dynamic transitions from adolescence to adulthood. Expanding research beyond student populations will also be essential to improve the generalizability and societal relevance of findings.

## Data Availability

The raw data supporting the conclusions of this article will be made available by the authors, without undue reservation.
